# Association between Skin Carotenoid Levels and Cognitive Impairment Screened by Mini-Cog in Patients with Glaucoma

**DOI:** 10.3390/cimb46070413

**Published:** 2024-07-03

**Authors:** Yuji Takayanagi, Yoichi Kadoh, Junichi Sasaki, Akira Obana, Masaki Tanito

**Affiliations:** 1 Department of Ophthalmology, Faculty of Medicine, Shimane University, Izumo 693-8501, Shimane, Japan; y.takayanagi1008@gmail.com (Y.T.); ykadoh@med.shimane-u.ac.jp (Y.K.);; 2 Department of Ophthalmology, Seirei Hamamatsu General Hospital, Hamamatsu 430-8558, Shizuoka, Japan; obana@sis.seirei.or.jp

**Keywords:** carotenoids, Veggie Meter, glaucoma, cognitive function, Mini-Cog

## Abstract

Carotenoids, having strong antioxidant properties, have been associated with neurodegenerative conditions like dementia and glaucoma, characterized by neuronal loss leading to cognitive and visual dysfunction. Therefore, carotenoids have attracted attention as factors predictive of the onset and progression of these neurodegenerative diseases. However, the impact of carotenoids on cognitive impairment and glaucomatous visual field defects remains unexplored. We conducted a retrospective, observational clinical study to investigate the association between skin carotenoid (SC) levels and cognitive impairment, as screened by the Mini-Cog test, in glaucoma patients. The study included 406 participants and 812 eyes were examined (average age: 69.7 ± 11.4 years; 228 men, 178 women) with various types of glaucoma: primary open angle (57.6%), exfoliation (18.6%), and other types (23.8%). SC levels were estimated via pressure-mediated reflection spectroscopy. Mixed-effects regression models were utilized to examine the relationship between SC levels, visual field defects, and Mini-Cog results. Of the participants, 28 (6.9%) tested positive on the Mini-Cog, suggesting cognitive impairment. The average SC level in the Mini-Cog positive group was significantly lower than in the negative group (269.5 ± 86.4 A.U. vs. 329.2 ± 120.4 A.U., respectively; *p* = 0.01). Additionally, the visual field mean deviation (MD) in the Mini-Cog positive group was notably worse than that in the negative group (−19.64 ± 9.07 dB vs. −12.46 ± 9.28 dB, respectively; *p* < 0.0001). The mixed-effects regression analysis revealed a significant association between Mini-Cog positivity and lower SC levels (*p* = 0.0006), although SC levels did not significantly correlate with MD (*p* = 0.3). Our findings suggest that cognitive impairment in glaucoma patients is associated with lower SC levels, underscoring the potential benefits of maintaining carotenoid levels to slow cognitive function decline. The protective role of carotenoids in glaucoma merits further investigation.

## 1. Introduction

Carotenoids, organic pigments produced by plants and algae, are significant antioxidants known for quenching free radicals and inhibiting lipid peroxidation [1,2]. Owing to their robust antioxidant and anti-inflammatory properties, carotenoids are increasingly recognized for their potential protective roles in the pathogenesis of neurodegenerative disorders, including dementia and glaucoma. Both conditions are characterized by neuronal loss, leading to cognitive and visual dysfunction [3,4].

Glaucoma constitutes a spectrum of neurodegenerative ocular conditions, and its progression is known to be irreversible [5]. It is estimated that over 70 million people worldwide suffer from glaucoma, making it a leading cause of diminished vision and blindness globally, including in Japan [6,7]. The disease is characterized by increased oxidative stress, a result of excessive reactive oxygen species (ROS) and impaired antioxidant mechanisms. In glaucoma, mitochondrial dysfunction amplifies ROS production, resulting in inflammatory damage to the retinal ganglion cells (RGCs) [8]. The use of antioxidants has been shown to counteract the inflammation triggered by ROS and to enhance the condition of RGCs in glaucoma models [9]. The neurodegenerative impact of ROS is considered a modifiable aspect in the development and progression of glaucoma [10,11]. Our prior research found that lower serum biological antioxidant levels are associated with elevated intraocular pressure and more significant visual field loss in primary open-angle glaucoma [12,13]. Research on the effects of dietary carotenoid supplementation in controlled glaucoma trials has been conducted, yet no substantial results have been observed [14,15,16]. Despite the presumed significant role of carotenoids in glaucoma, their precise monitoring is currently not achievable.

While the link between dietary carotenoid intake and dementia prevalence remains a subject of debate, several studies have highlighted a significant association between carotenoid consumption, cognitive impairment, and a positive effect in preventing Alzheimer’s disease [17,18,19]. Alzheimer’s disease and other dementias are multifactorial disorders with various hypothesized pathophysiologies [20,21]. Recent research underscores the contribution of neuroinflammation to dementia’s pathogenesis [22,23], where synaptic dysfunction and neuronal death occur due to excessive inflammatory molecules. These molecules disrupt the blood–brain barrier and increase abnormal Amyloid beta protein production [24,25]. Hence, carotenoids are thought to play a crucial role in dementia pathogenesis through their antioxidant and anti-inflammatory capabilities [26].

Despite the presumed importance of carotenoids in neurodegenerative diseases, accurate and consistent monitoring remains a challenge. Percutaneous fingertip measurements offer a promising approach for multiple carotenoid assessments. This non-invasive, convenient method does not require blood sampling or complex procedures. Subjects simply place their fingers on the device for approximately 10 s. The skin carotenoid (SC) levels measured via pressure-mediated reflection spectroscopy (RS) correlate well with serum carotenoid levels determined by high-performance liquid chromatography (HPLC) [27]. The RS method is capable of measurements in the 350–850 nm range, encompassing the carotenoid absorption wavelengths around 480 nm. With RS methods, all chromophores in the skin including α- and β-carotenes, β-cryptoxanthin, lycopene, lutein, and zeaxanthin were taken into account in the calculation of a composite score [27]. Additionally, studies have shown only a weak correlation between skin melanin content and carotenoids, suggesting that SC levels are not significantly affected by melanin absorption [27]. Several studies have shown the usefulness of SC evaluation by RS in large clinical studies [28,29,30].

The Mini-Cog cognitive function test, a brief cognitive screening test, comprises a 3-item word recall and a clock drawing test [31]. The Mini-Cog, scored by an algorithm as “possibly impaired (score ≤ 2)” or “probably normal (score ≥ 3),” and the Mini-Mental State Examination (MMSE), at a cut-point of 25, exhibited similar sensitivity (76% vs. 79%) and specificity (89% vs. 88%) for dementia [31]. In this context, we conducted a retrospective, observational clinical study to investigate the relationship between SC levels and cognitive impairment, as assessed by the Mini-Cog test, in patients with glaucoma. This study aimed to investigate the relationships between carotenoids and cognitive function in glaucoma patients. We believe this study highlights the potential link between carotenoids and neurodegenerative diseases, focusing on cognitive aspects in glaucoma patients.

## 2. Materials and Methods

### 2.1. Subjects

This study adhered to the tenets of the Declaration of Helsinki and was approved by the institutional review board (IRB) of Shimane University Hospital (IRB No. 20200228-2, issued on 21 June 2021). All participant information was anonymized. We conducted a retrospective analysis including all glaucoma patients who underwent Mini-Cog score and SC score measurements at the outpatient clinics of the hospitals. Patients with fundus diseases affecting visual acuity other than glaucoma and cataract were excluded. Our study population consisted of 812 eyes from 406 Japanese participants (228 men, 178 women; mean age ± SD, 79.5 ± 7.6 years), including subjects with primary open-angle glaucoma (57.6%), exfoliation glaucoma (18.6%), and others (23.8%). We collected data from medical charts including age, sex, current smoking status, body mass index (BMI), systolic and diastolic blood pressure (BP), heart rate (HR), SC levels, cognitive function (rated from 0 (poor) to 5 (good) using the Mini-Cog test), and ophthalmologic measurements such as best corrected visual acuity (VA), highest intraocular pressure (IOP), lens status (phakia or pseudophakia/aphakia), number of antiglaucoma medications, mean deviation (MD) of the visual field (Central 30-2 Program, Humphrey Visual Field Analyzer, Carl Zeiss Meditec, Dublin, CA, USA), and glaucoma types. We conducted the Mini-Cog test and SC score measurements on the first visit. If patients were not communicative, the Mini-Cog test was not conducted.

### 2.2. Measurement of Skin Carotenoid Levels

SC levels were measured using pressure-mediated RS (Veggie Meter^®^, Longevity Link Corporation, Salt Lake City, UT, USA). The principles of this device are detailed elsewhere [27]. Measurements followed the device manufacturer’s instructions. Calibration with manufacturer-provided reference materials was carried out prior to daily skin measurements, conducted once daily (before morning sessions). For SC measurement, subjects inserted their left middle finger into the device’s cradle, and the SC index was calculated as the average of three consecutive readings for each subject.

### 2.3. Statistical Analysis

The subjects were divided into 2 groups based on the cognitive impairment screening positive (Mini-Cog score, ≤2) and negative (≥3). For group comparisons, continuous data differences were analyzed using the unpaired Student t-test, and categorical data differences were examined using Fisher’s exact probability test. To identify independent factors related to SC levels and MD, mixed-effects regression models were employed. In one model, SC levels were the response variable, with each patient’s ID number as a random effect, and factors such as age, sex, smoking status, BMI, mean BP, HR, pseudoexfoliation presence, lens status (phakia or pseudophakia), history of any intraocular surgery, VA, IOP, number of antiglaucoma medications, MD, and Mini-Cog results as fixed effects. Another model used MD as the response variable, incorporating the same factors plus SC levels. Statistical analyses were performed using JMP Pro statistical software version 14.2 (SAS Institute, Inc., Cary, NC, USA). All *p*-values are two-sided. Data are presented as means ± SD for continuous variables and as numbers and percentages for categorical variables. Decimal VAs were converted to the logarithm of the minimum angle of resolution (LogMAR) for statistical analysis. VAs such as counting fingers, hand motions, light perception, and no light perception were assigned decimal VAs of 0.0025, 0.002, 0.0016, and 0.0013, respectively. The dataset underlying this study is found in Supplementary Table S1.

## 3. Results

The demographic subject data, including age, sex, presence of current smoking habit, BMI, mean blood pressure, heart rate, Mini-Cog score, and SC index, are shown in Table 1. Twenty-eight of the 406 participants (6.9%) were Mini-Cog positive, i.e., suspect of cognitive impairment. The mean carotenoids levels were 325.1 ± 119.3 in the study participants.

Table 2 shows the characteristics of subjects stratified by Mini-Cog results. The mean SC level of the Mini-Cog positive group was lower than that of Mini-Cog negative group (*p* = 0.01). Compared to the Mini-Cog negative group, a higher mean age was also observed in the Mini-Cog positive group. The other parameters, including sex, current smoking habit, BMI, mean BP, and HR, were not significantly different between the two groups.

Table 3 summarizes eye-based ophthalmologic data of individuals stratified by Mini-Cog results. The MD of the Mini-Cog positive group was significantly worse than that of the Mini-Cog negative group (*p* < 0.0001). Compared to the Mini-Cog negative group, worse VA, higher IOP, and more pseudophakia or aphakia were found in the Mini-Cog positive group. The presence of pseudoexfoliation, the number of antiglaucoma medications, and the types of glaucoma were not significantly different between the two groups.

**Figure 1:**
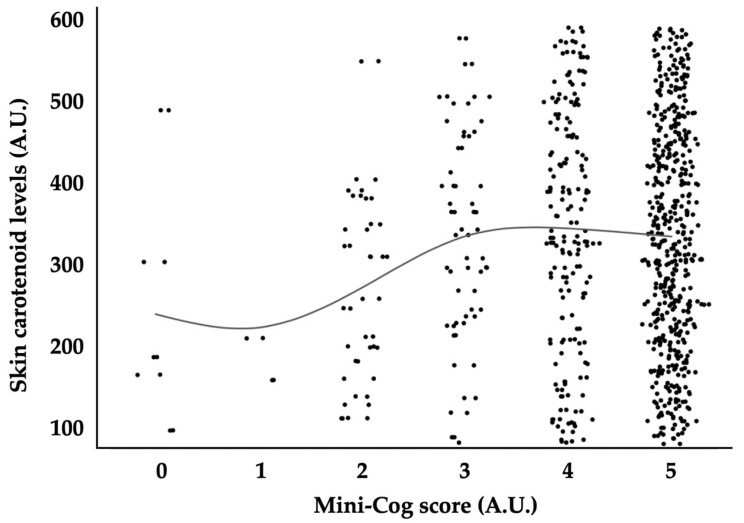
Relationships between Mini-Cog score and skin carotenoid levels. A.U., arbitrary unit. Solid line indicates a moving average.

Table 4 demonstrates mixed-effects regression models with skin carotenoid levels as the response variable. The model indicated that sex, current smoking habit, heart rate, and Mini-Cog result were independent variables significantly associated with skin carotenoids levels (*p* < 0.0001, *p* = 0.0001, *p* = 0.0002, and *p* = 0.0006, respectively). The other parameters, including age, BMI, mean BP, the presence of pseudoexfoliation, lens status, the history of post intraocular surgery, VA, IOP, the number of antiglaucoma medications, and MD, were not significantly associated with skin carotenoid levels (*p* = 0.5, *p* = 0.9, *p* = 0.3, *p* > 0.9, *p* > 0.9, *p* > 0.9, *p* > 0.9, *p* > 0.9, *p* > 0.9, and *p* > 0.9, respectively). The distribution of SC levels in relation to Mini-Cog scores is depicted in Figure 1.

**Figure 2:**
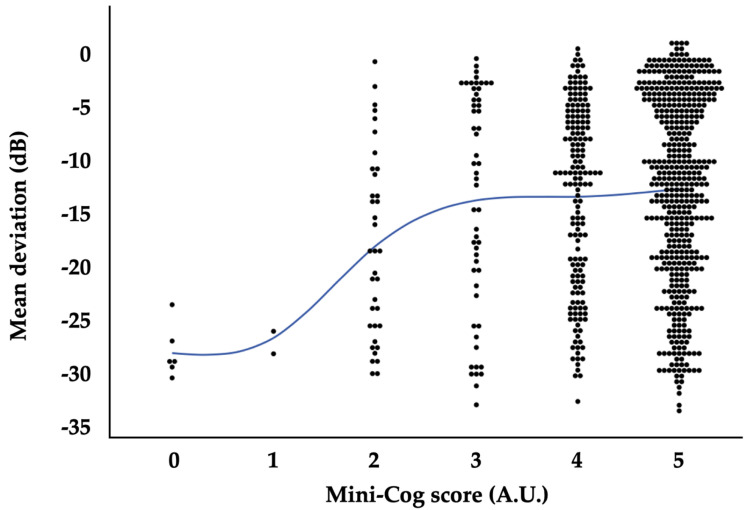
Relationships between Mini-Cog score and visual field mean deviation. A.U., arbitrary unit; dB, decibels. Solid line indicates a moving average.

Table 5 reveals mixed-effects regression models with MD as the response variable. The model indicated that lens status, the history of intraocular surgery, VA, IOP, the number of antiglaucoma medications, and Mini-cog result were independently associated with MD (*p* < 0.0001, *p* = 0.04, *p* < 0.0001, *p* = 0.009, *p* < 0.0001, and *p* = 0.005, respectively). However, SC level was not significantly associated with the visual filed MD (*p* = 0.3). The other parameters, including age, sex, current smoking habit, BMI, mean BP, HR, and the presence of pseudoexfoliation, were also not significantly associated with the visual filed MD (*p* = 0.2, *p* = 0.3, *p* = 0.7, *p* > 0.9, *p* = 0.3, *p* = 0.3, and *p* = 0.4, respectively). The distribution of MD in relation to Mini-Cog scores is illustrated in Figure 2.

## 4. Discussion

This study aimed to explore potential relationships between carotenoids and cognitive function in glaucoma patients. Our findings offer two key clinical insights. First, we observed that the mean SC level was lower in the Mini-Cog positive group compared to the Mini-Cog negative group, with a significant association between Mini-Cog positivity and lower SC levels. Second, while the MD in the Mini-Cog positive group was significantly worse than in the Mini-Cog negative group, we found no significant association between SC levels and visual field MD.

First, our findings revealed that being Mini-Cog positive was independently associated with lower SC levels. Specifically, the mean SC level was significantly lower in patients who were Mini-Cog positive, suggesting cognitive impairment, compared to those who were Mini-Cog negative. Previous reports identified significant associations between lower cognitive performance and certain serum carotenoids [17]. Additionally, a meta-analysis found a significant effect of carotenoid intervention on cognitive outcomes [18]. Our results support the existing relationship between carotenoids and cognitive function in glaucoma patients. A possible explanation for the protective roles of carotenoids in cognitive function lies in their antioxidant and anti-inflammatory properties [19,26,32]. Research has shown that carotenoids can interact with the nuclear factor κB pathway, inhibiting the production of inflammatory cytokines such as interleukins and prostaglandins [32]. Carotenoids are also known to activate the Nrf2-antioxidant response element signaling pathway, regulating the expression of genes involved in the detoxification and elimination of reactive oxidants and electrophilic agents, thereby enhancing cellular antioxidant capacity [33]. These inflammatory and oxidative molecules are known to disrupt the integrity of the blood–brain barrier and increase the production of Aβ peptide, implicated in the pathogenesis of neurodegenerative disorders like Alzheimer’s disease. Therefore, it is biologically plausible that carotenoids contribute to better cognitive performance.

The second clinical observation that we presented is that SC levels did not show a significant association with visual field MD in glaucoma patients. Previous research highlighted the neuroprotective effects of carotenoids in eyes affected by glaucoma [3,34]. Clinical studies have indicated a protective trend linking higher dietary carotenoid intake with a reduced risk of glaucoma, and increased carotenoid levels in macular pigment have been associated with improved visual performance in glaucomatous eyes [3,35]. Notably, lutein has been shown to provide enhanced neuroprotection, supporting retinal ganglion cell survival and preserving synaptic activity [36]. However, the relationship between carotenoid consumption and the progression of glaucomatous visual field defects has been less explored. Our study was unable to confirm this association. One reason for the lack of a significant association between MD and SC level is that MD is influenced by factors such as IOP, the duration of glaucoma, and treatment regimen, potentially obscuring the neuroprotective effects of carotenoids. The protective effects for MD by carotenoids needs further research.

In this study, the MD in the Mini-Cog positive group was significantly worse than in the Mini-Cog negative group. The mixed-effects regression models revealed a significant relationship between MD and Mini-Cog scores. It remains challenging to discern whether the worsened MD in the Mini-Cog positive group is due to cognitive impairment or more severe glaucomatous damage. Our previous research indicated that reduced cognitive function, as screened by Mini-Cog, was a risk factor for unsuccessful eyedrop instillation [37]. This could lead to poorer IOP control, potentially explaining the worse MD observed in the Mini-Cog positive group. However, our current study did not take into account visual field reliability factors such as false positives (FP) and false negatives (FN). We previously reported an association between abnormal Mini-Cog scores and increased rates of FN and FP, particularly noting that lower word recall test scores were linked to higher FN rates [38]. These factors could influence the interpretation of our current results and thus warrant further investigation.

It is noteworthy that significantly higher carotenoid levels were observed in women and non-smokers. This correlation between SC levels, sex, and smoking status mirrors findings in diabetic patients [29]. The trend of higher carotenoid levels in women and non-smokers is well-documented [30,39]. This could be attributed to the reported higher dietary intake of carotenoids among women compared to men [40], suggesting that dietary habits might underpin these correlations. Additionally, HR emerged as an independent variable significantly associated with SC levels. Given the established protective effects of carotenoids against cardiovascular diseases [41,42,43], this observed correlation could reflect a potential reduction in cardiovascular events linked to higher dietary carotenoid consumption.

Lastly, it is crucial to acknowledge several limitations in our study that may impact the generalizability of our findings. First, as with any retrospective study, ours was neither controlled nor randomized, which is a common limitation in such research designs. Second, the inclusion of only glaucomatous patients introduces potential selection bias. We defined controls that were participants with glaucoma and Mini-Cog negative results, allowing for reasonable comparisons. However, we have to explore the data in participants without glaucoma who underwent the Mini-Cog test and SC measurements. Third, our study assessed total carotenoid levels in the skin, encompassing both xanthophyll carotenoids and carotenes, which could influence the interpretation of our results. We can measure the types of carotenoids by taking blood samples, but it is an invasive and time-consuming method for participants. Therefore, we only measured skin carotenoid levels by the Veggie Meter non-invasively. Lastly, we did not evaluate the dairy intake of carotenoids. This might affect the interpretation of this study. Despite these limitations, our study boasts several strengths, including a large sample size, the non-invasive and objective measurement of SC levels, and the comprehensive assessments of patients’ clinical characteristics. To our knowledge, this is the first study to explore in detail the association between SC levels and cognitive function as assessed by the Mini-Cog test.

## 5. Conclusions

In conclusion, our study found that cognitive impairment, as assessed by Mini-Cog, is associated with lower SC levels in glaucoma patients. This highlights the potential benefits of maintaining carotenoid levels to mitigate cognitive function deterioration. The protective role of carotenoids in glaucoma deserves further exploration.

## Figures and Tables

**Table 1:** Patient characteristics of the study participants.

N	406
Age (years)	
Mean ± SD	69.7 ± 11.4
range	34, 92
Sex	
Men, n (%)	228 (56.2)
Women, n (%)	178 (43.8)
Current smoking	
Yes, n (%)	51 (12.7)
No, n (%)	352 (87.3)
BMI (kg/m^2^)	
Mean ± SD	23.2 ± 3.5
range	12.3, 37.8
Mean BP (mmHg)	
Mean ± SD	101.9 ± 14.1
range	70.0, 148.3
HR (bpm)	
Mean ± SD	74.1 ± 12.2
range	49, 117
Mini-Cog score	
5, n (%)	254 (62.6)
4, n (%)	93 (22.9)
3, n (%)	31 (7.6)
2, n (%)	21 (5.2)
1, n (%)	2 (0.5)
0, n (%)	5 (1.2)
positive, n (%)	28 (6.9)
negative, n (%)	378 (93.1)
Skin carotenoid (A.U.)	
Mean ± SD	325.1 ± 119.3
range	78.0, 803.5

Data are presented as mean ± SD or as percentages. Mini-Cog score is presented as total score of the recall test graded on a scale of 0 to 3 and the clock drawing test graded on 0 or 2. A score of 0–2 indicates a positive dementia screen. A score of 3–5 indicates a negative dementia screening. N, number of participants; SD, standard deviation; BMI, body mass index; kg/m^2^, kilogram per square meter; BP, blood pressure; mmHg, millimeter of mercury; HR, heart rate; bpm, beat per minute; A.U., arbitrary unit.

**Table 2:** Characteristics of subjects stratified by positive/negative of Mini-Cog.

	Positive	Negative	*p*-Value ^a^
N	28	378	
Age (years)			
Mean ± SD	79.5 ± 7.6	69.0 ± 11.3	<0.0001 **
range	64, 90	34, 92	
Sex			
Men, n (%)	17 (60.7)	211 (55.8)	0.7
Women, n (%)	11 (39.3)	167 (44.2)	
Current smoking			
Yes, n (%)	1 (3.6)	50 (13.3)	0.23
No, n (%)	27 (96.4)	320 (86.7)	
BMI (kg/m^2^)			
Mean ± SD	23.5 ± 3.4	23.2 ± 3.5	0.6
range	17.4, 29.6	12.3, 37.8	
Mean BP (mmHg)			
Mean ± SD	91.9 ± 30.4	100.8 ± 18.1	0.1
range	70.7, 132.7	70.0, 148.3	
HR (bpm)			
Mean ± SD	74.3 ± 14.5	74.0 ± 12.0	0.9
range	49, 105	50, 117	
Skin carotenoid (A.U.)			
Mean ± SD	269.5 ± 86.4	329.2 ± 120.4	0.01 *
range	136.5, 527.5	78.0, 803.5	

^a^ Comparisons between the two groups divided by Mini-Cog results by using unpaired Student t-test for continuous data and by using Fisher’s exact probability test for categorical data. The * and ** correspond to the significance levels at 5% (*p* < 0.05) and 1% (*p* < 0.01), respectively. N, number of participants; SD, standard deviation; BMI, body mass index; kg/m^2^, kilogram per square meter; BP, blood pressure; mmHg, millimeter of mercury; HR, heart rate; bpm, beat per minute; A.U., arbitrary unit.

**Table 3:** Eye-based ophthalmologic data of individuals stratified by positive/negative of Mini-Cog.

	Positive	Negative	*p*-Value ^a^
N	56	756	
VA (LogMAR)			
Mean ± SD	0.61 ± 0.86	0.24 ± 0.54	<0.0001 **
range	−0.08, 2.89	−0.08, 2.89	
IOP (mmHg)			
Mean ± SD	24.0 ± 12.0	21.4 ± 8.9	0.04 *
range	9, 59	6, 76	
Lens status			
Phakia	17 (30.4)	401 (53.0)	0.001 **
Pseudophakia/aphakia	39 (69.6)	355 (47.0)	
Pseudoexfoliation			
Yes, n (%)	17 (30.4)	144 (19.1)	0.054
No, n (%)	39 (69.6)	612 (80.9)	
Antiglaucoma medications (n)			
Mean ± SD	2.4 ± 1.4	2.6 ± 1.4	0.4
range	0, 5	0, 6	
MD (dB)			
Mean ± SD	−19.64 ± 9.07	−12.46 ± 9.28	<0.0001 **
range	−30.67, 0.25	−33.89, 1.84	
Types of glaucoma			
POAG, n (%)	30 (53.6)	438 (57.9)	0.1
EXG, n (%)	16 (28.6)	135 (17.9)	
Others, n (%)	10 (17.9)	183 (24.2)	

^a^ Comparisons between the two groups divided by Mini-Cog results by using unpaired Student t-test for continuous data and by using Fisher’s exact probability test for categorical data. The * and ** correspond to the significance levels at 5% (*p* < 0.05) and 1% (*p* < 0.01), respectively. N, number of participants; SD, standard deviation; VA, visual acuity; LogMAR, logarithm of the minimum angle of resolution; IOP, intraocular pressure; mmHg, millimeter of mercury; n, number of medications; MD, mean deviation; dB, decibel; POAG, primary open angle glaucoma; EXG, exfoliation glaucoma.

**Table 4:** Mixed-effects regression models for skin carotenoid levels.

	Estimate	Lower CI	Upper CI	*p* Value ^a^
Age (/year)	0.29	−0.60	1.175	0.5
Women (/men)	23.08	14.12	32.05	<0.0001 **
Current smoking (/no)	−25.90	−39.21	−12.60	0.0001 **
BMI (/kg/m^2^)	−0.20	−2.91	2.51	0.9
Mean BP (/mmHg)	−0.37	−1.06	0.33	0.3
HR (/bpm)	−1.53	−2.33	−0.73	0.0002 **
Pseudoexfoliation (/no)	0.01	−0.01	0.01	>0.9
Phakia (/pseudophakia or aphakia)	−0.01	−0.01	0.01	>0.9
Post intraocular surgery (/no)	−0.01	−0.01	0.01	>0.9
VA (/LogMAR)	−0.01	−0.01	0.01	>0.9
IOP (/mmHg)	0.01	−0.01	0.01	>0.9
Antiglaucoma medications (/number)	0.01	−0.01	0.01	>0.9
MD (/dB)	0.01	−0.01	0.01	>0.9
Mini-Cog (/negative)	−32.07	−50.40	−13.74	0.0006 **

^a^ Mixed effects regression models were performed with skin carotenoid levels as the response variable in which each patient’s identification number was regarded as a random effect, and the following factors were regarded as a fixed effect: age, sex, the presence of current smoking habit, BMI, mean BP, HR, the presence of pseudoexfoliation, lens status, any history of intraocular surgery, VA, highest IOP, the number of antiglaucoma medications, MD, the results of Mini-Cog. The ** corresponds to the significance level at 1% (*p* < 0.01). CI, confidence interval; BMI, body mass index; BP, blood pressure; HR, heart rate; VA, visual acuity; LogMAR, logarithm of the minimum angle of resolution; mmHg, millimeter of mercury; MD, mean deviation.

**Table 5:** Mixed-effects regression models for MD.

	Estimate	Lower CI	Upper CI	*p* Value ^a^
Age (/year)	0.042	−0.03	0.11	0.2
Women (/men)	0.38	−0.34	1.10	0.3
Current smoke (/no)	−0.24	−1.31	0.83	0.7
BMI (/kg/m^2^)	0.01	−0.20	0.20	>0.9
Mean BP (/mmHg)	0.03	−0.02	0.08	0.3
HR (/bpm)	0.03	−0.03	0.09	0.3
Pseudoexfoliation (/no)	−0.33	−1.17	0.51	0.4
Phakia (/pseudophakia or aphakia)	−1.67	−2.41	−0.92	<0.0001 **
Post intraocular surgery (/no)	−1.10	−2.13	−0.06	0.04 *
VA (/LogMAR)	−8.23	−9.53	−6.94	<0.0001 **
IOP (/mmHg)	−0.10	−0.17	−0.02	0.009 *
Antiglaucoma medications (/number)	−1.29	−1.76	−0.82	<0.0001 **
Mini-Cog (/negative)	−2.14	−3.60	−0.66	0.005 **
Skin carotenoid (/A.U.)	0.01	−0.01	0.01	0.3

^a^ Mixed effects regression models were performed with MD as the response variable in which each patient’s identification number was regarded as a random effect, and the following factors were regarded as a fixed effect: age, sex, the presence of current smoking habit, BMI, mean BP, HR, the presence of pseudoexfoliation, lens status, any history of intraocular surgery, VA, highest IOP, the number of antiglaucoma medications, the results of Mini-Cog, skin carotenoid. The * and ** correspond to the significance levels at 5% (*p* < 0.05) and 1% (*p* < 0.01), respectively. MD, mean deviation; CI, confidence interval; BMI, body mass index; BP, blood pressure; HR, heart rate; VA, visual acuity; LogMAR, logarithm of the minimum angle of resolution; mmHg, millimeter of mercury; A.U., arbitrary unit.

## Data Availability

The data presented in this study are available in this manuscript.

## Supplementary Materials

The following supporting information can be downloaded at: https://www.mdpi.com/article/10.3390/cimb46070413/s1; Supplementary Table S1.
