# Supplementary material for: Association between Skin Carotenoid Levels and Cognitive Impairment Screened by Mini-Cog in Patients with Glaucoma
Source: Curr Issues Mol Biol. 2024 Jul 3;46(7):6940–50. doi: 10.3390/cimb46070413 (PMC11275399; doi:10.3390/cimb46070413)
Supplement: Supplementary file 1 [file cimb-46-00413-s001.zip › cimb-3057449-supplementary.pdf]

Study ID	age	sex	SC	smoking	BMI	bp	pulse	word	clock	Mini-Cog	lensr	lensl	exr	exl	glar	glal	var	val	iopr	iopl	medr	medl	mdr	mdl
2	70	M	350	0	22.23896	70.66667	64	0	2	2	lens	IOL	n	n	POAG	POAG	0.30103	0.154902	25	55	5	5	-25.55	-27.93
7	49	F	475	0		123.6667	93	3	2	5	lens	lens	n	n	POAG	POAG	0.045757	-0.07918	22	23	0	0	-3.04	-4.43
9	62	M	248	0	24.21229	120	75	3	2	5	lens	lens	n	n	POAG	POAG	-0.07918	-0.07918	21	17	4	4	-29.91	-22.97
10	82	M	275.5	0	19.48696	122.6667	67	3	2	5	lens	lens	n	n	other	other	0.39794	2	21	22	3	3	-6.92	-23.87
21	76	F	482.5		24.03461	92.33333	70	2	2	4	IOL	IOL	n	n	other	other	-0.07918	-0.07918	25	9	3	0	-8.61	-1.8
23	70	F	244.5	0	20.50493	98.33333	77	3	2	5	IOL	IOL	n	n	POAG	POAG	0.045757	-0.07918	19	19	4	4	-8.15	-6.84
24	66	F	240.5	0	20.44444	74	66	2	2	4	IOL	IOL	n	n	POAG	POAG	0.154902	1.09691	17	17	3	0	-18.07	-19.7
25	73	M	130	0	18.06617	92.33333	87	3	2	5	lens	lens	n	n	POAG	POAG	-0.07918	0.154902	26	15	1	1	-13.81	-25.94
26	70	F	539.5	0	20.71979	95	67	2	2	4	lens	lens	y	y	EXG	EXG	0.154902	0.221849	15	13	3	3	-27.75	-21.4
43	71	F	345	1	25.95049	99	65	2	2	4	IOL	IOL	n	n	POAG	POAG	0.09691	-0.07918	17	17	5	4	-28.79	-2.41
44	53	M	297	0		101.3333	86	3	2	5	lens	lens	n	n	POAG	POAG	-0.07918	-0.07918	15	13	2	2	-11.44	-17.2
62	77	F	442	0	28.80778	112.3333	83	3	2	5	IOL	IOL	n	n	POAG	POAG	2.60206	0.09691	8	15	3	1		-11.24
63	72	M	320	0	23.30856	113	61	3	2	5	IOL	IOL	n	n	POAG	POAG	0.154902	0.522879	43	17	3	0	-28.75	-22.27
65	82	M	489.5	0	20.39591	111	72	3	2	5	IOL	IOL	y	y	EXG	EXG	0	0	19	31	4	4	0.24	-13.56
66	77	F	245.5	0	24.88627	103	58	3	2	5	IOL	IOL	y	n	EXG	other	0.522879	0.39794	19	16	2	2	-22.38	-26.07
71	85	M	234.5	0	21.35991	120.6667	55	2	0	2	IOL	IOL	y	y	EXG	EXG	0.221849	2.69897	23	25	3	3	-18.25	-23.87
77	72	F	527	0	18.54935	88.66667	83	3	2	5	lens	lens	y	y	EXG	EXG	0.30103	1.30103	14	28	5	5	-7.32	-25.84
84	62	F	165	0	21.50189	96	109	2	2	4	lens	lens	n	n	other	other	1.154902	0.39794	24	19	1	1		
86	56	M	324.5	0	24.80159	92.66667	62	3	2	5	IOL	IOL	n	n	other	other	2.886057	0		19	0	4		-4.03
88	76	M	299	0	19.4674	103.3333	70	0	2	2	lens	IOL	n	n	POAG	POAG	0.154902	2	29	23	3	0	-10.47	-23
98	67	F	306.5	0	27.47624	128	72	2	2	4	lens	lens	n	n	POAG	POAG	0	0.045757	22	22	1	1	-1.67	-3.22
99	75	M	432	0	20.27344	99	68	3	2	5	IOL	IOL	y	n	other	other	2.60206	1.522879	8	15	1	1	-29.96	-24.27
100	58	M	410	1	24.56747	111.3333	74	3	2	5	lens	lens	n	n	POAG	POAG	0	0.69897	31	20	3	3	-13.91	-16.24
101	80	M	437.5	0	25.55885	116.6667	71	3	2	5	lens	lens	n	n	POAG	POAG	0.522879	0.39794	43	26	3	3	-9.91	-5.35
102	71	M	209.5	0	29.72108	92.66667	69	3	2	5	IOL	IOL	n	n	POAG	POAG	0	0.154902	19	17	3	3	-4.4	-20.39
108	76	M	532.5	0	28.04038	112	72	2	2	4	IOL	IOL	n	y	POAG	EXG	0.221849	0.30103	21	33	3	3		
109	86	M	412.5	0	24.34961	94.33333	78	3	2	5	IOL	IOL	y	y	EXG	EXG	0.30103	0.045757	23	35	3	0	-3.61	-13.72
110	85	F	400	0	28.06183	129.6667	104	2	2	4	IOL	IOL	n	y	POAG	EXG	-0.07918	0.045757	17	18	0	0	-18.94	-10.46
111	63	M	188.5	1	21.54509	119	87	3	2	5	IOL	IOL	n	n	POAG	POAG	0	0	25	20	3	3	-20.05	-10.36
112	84	F	520.5		22.98743	109	70	3	2	5	IOL	IOL	n	n	other	other	0.30103	0.30103	19	21	3	3	-11.77	-13.39
113	70	M	233	0	28.60476	100	85	3	2	5	IOL	IOL	n	n	POAG	POAG	-0.07918	0.30103	16	16	3	3	0.39	-18.56
114	59	F	402	0	28.19428	126.3333	54	2	2	4	IOL	IOL	n	n	other	other	0	0.045757	19	19	3	3	-4.04	-11.08
115	62	F	344	0	26.56434	100	91	3	2	5	IOL	IOL	y	y	EXG	EXG	0.154902	1.39794	18	24	0	0	-28.34	-29.42
128	74	F	304	0	28.9912	107.6667	64	2	2	4	lens	lens	n	n	other	other	0.09691	0	21	22	5	5	-7.3	-12.18
130	79	M	384.5	0	25.79617	114.3333	88	3	2	5	IOL	IOL	y	y	EXG	EXG	0	0	27	31	3	3	-23.32	-14.92
137	74	F	306	0	16.9225	122	63	3	2	5	IOL	IOL	n	n	POAG	POAG	-0.07918	-0.07918	14	15	3	3	-9.49	-8.53
138	72	F	285	0	24.88542	95.33333	81	2	2	4	IOL	IOL	n	n	POAG	POAG	0.69897	0.221849	21	15	0	2	-19.69	-3.57
143	81	F	293	0	33.17758	113	99	2	2	4	IOL	IOL	n	n	other	other	0.39794	0.221849	19	20	3	3	-4.39	-6.38
161	54	F	444	0	19.10328	91	66	2	2	4	lens	lens	n	n	other	other	0.09691	0.30103	22	56	5	5	-3.25	-26.31
164	86	F	362.5	0	22.53537	90.66667	84	1	2	3	lens	lens	n	n	POAG	POAG	0.30103	0	6	9	3	1	-14.06	-1.77
166	63	M	255.5	0	19.76514	100.6667	80	3	0	3	lens	lens	n	n	POAG	POAG	0.69897	0.39794	23	22	3	3	-4.16	-3.54
167	86	M	117.5	0	15.58451	105.6667	65	1	2	3	IOL	IOL	n	n	POAG	POAG	2.69897	0.69897	41	19	4	4	-30.35	-25.73
168	82	F	374.5	0	19.96528	87.33333	79	3	2	5	lens	lens	n	y	POAG	EXG	1.045757	0.69897	13	18	4	4		
170	76	M	394	1	24.47289	103.3333	62	3	2	5	IOL	lens	n	n	other	POAG	0.045757	0	34	27	3	3	-27.11	-2.45
179	67	M	285	1	26.38213	108	67	3	0	3	IOL	lens	y	n	EXG	other	0.045757	0	58	21	2	0	-1.35	-1.84
180	62	M	248	0	25.43006	118.3333	97	3	2	5	lens	lens	n	n	other	other	0	0	38	32	4	4	-29.91	-22.97
184	77	M	273.5	0	27.81145	91.66667	84	1	2	3	lens	lens	y	y	EXG	EXG	0.045757	-0.07918	26	23	5	2	-29.8	0.55
185	79	F	325	0	20.52211	105	79	3	2	5	IOL	IOL	y	y	EXG	EXG	0.69897	0.09691	48	14	4	0	-13.12	-3.38
193	81	M	296	0	23.23346	136	90	2	2	4	lens	lens	n	n	other	other	0.30103	0	22	25	3	3	-4.69	-3.82
194	69	F	380.5	0	22.83288	118	69	2	2	4	lens	lens	n	n	POAG	POAG	0.30103	0.39794	20	22	3	3	-5.09	-12.18
195	70	F	470.5	0	24.7768	120	92	3	2	5	lens	lens	n	n	POAG	POAG	0.154902	0.39794	21	20	3	3	-19.57	-15.78
196	63	F	214	1		108.3333	72	3	2	5	lens	lens	n	n	POAG	POAG	-0.07918	-0.07918	17	17	3	3	-18.13	-19.23
199	79	M	606	0	20.56881	87.66667	79	3	2	5	IOL	IOL	y	y	EXG	EXG	0.154902	-0.07918	29	14	3	1	-18.88	0.31
200	47	F	594.5	0	22.81949	116.3333	71	3	2	5	IOL	lens	n	n	POAG	POAG	-0.07918	-0.07918	76	22	2	2	0.17	-0.77
202	72	F	603	0	20.80514	96.33333	76	1	2	3	IOL	IOL	n	y	POAG	EXG	-0.07918	2.60206	17	47	1	4	-2.17	-29.47
203	87	M	340	0	23.99946	120.6667	72	2	2	4	IOL	IOL	n	n	POAG	POAG	2.79588	0.045757	19	24	0	3		-27.64
209	70	F	520	0		111	75	3	2	5	IOL	IOL	n	y	POAG	EXG	0.09691	0.221849	18	21	3	4	-17.02	-21.31
210	74	F	514.5	0	20	103.3333	69	2	2	4	IOL	IOL	y	y	EXG	EXG	-0.07918	-0.07918	24	37	4	4	-0.61	-20.22
211	86	M	405	0	23.58833	125.6667	62	1	2	3	lens	lens	n	n	POAG	POAG	0.522879	0.30103	14	14	4	4	-11.82	-9.89
216	86	F	383.5	0	18.66667	86.33333	76	2	2	4	IOL	IOL	n	n	POAG	POAG	-0.07918	0.045757	22	35	3	3	-6.37	-29.15
223	66	M	238	0	26.81359	115.3333	90	3	2	5	lens	lens	n	n	POAG	POAG	-0.07918	2.69897	16	16	4	0	-2.3	-17.97
224	83	F	345.5	0	22.21297	126	105	2	0	2	lens	lens	y	y	EXG	EXG	0.69897	1.221849	13	31	5	5	-27.12	-29.19
226	64	F	355.5	0		97.33333	68	3	2	5	lens	lens	n	n	POAG	POAG	0	-0.07918	19	19	3	3	-3.43	-3.46
228	69	M	218.5	0	28.84153	106.3333	77	1	2	3	IOL	IOL	n	n	POAG	POAG	0.39794	1	34	42	0	2	-26.67	-29.56
237	69	F	514	0		99.33333	62	2	2	4	lens	lens	n	n	POAG	POAG	-0.07918	0.30103	31	31	3	3	-5.53	-21.9
238	85	F	445.5	0	23.42237	92	61	2	2	4	lens	lens	n	n	POAG	POAG	0.39794	0.39794	12	13	1	1		
240	87	F	429.5	0	30.29778	100.3333	74	2	2	4	IOL	lens	n	n	other	other	0.09691	0.221849	30	45	0	1	-25.22	-22.42

241	66	M	213	0	25.03992	113	99	3	2	5	lens	lens	y	y	EXG	EXG	0.221849	0.09691	22	20	3	3	-21.3	-2.7
242	71	M	304.5	0	26.66667	89	81	3	2	5	IOL	IOL	y	y	EXG	EXG	0.39794	0.045757	14	14	2	2	-10.49	-18.71
243	71	F	321.5	1	25.29938	100.3333	69	2	2	4	IOL	IOL	n	n	other	other	0.221849	-0.07918	17	21	2	2	-24.33	-4.55
244	64	F	794	0	27.40766	95.66667	91	3	2	5	lens	IOL	n	n	POAG	POAG	-0.07918	-0.07918	21	17	2	2	-0.52	-10.19
248	66	F	240.5	0	20.67346	86.66667	74	2	2	4	IOL	IOL	n	n	POAG	POAG	0.221849	1.09691	17	17	3	0	-17.24	-20.99
253	71	M	349.5	0	21.03141	105.3333	76	2	2	4	IOL	IOL	n	n	POAG	POAG	0.09691	0	45	42	0	4	-5.4	-7.07
255	61	M	252	1	28.9498	109.6667	89	3	2	5	IOL	IOL	n	n	POAG	POAG	0.30103	0.69897	30	21	4	4		-9.93
256	85	M	148.5	1	25.98638	93	75	2	2	4	IOL	IOL	y	n	EXG	POAG	2.886057	0	34	26	0	2		-16.04
258	79	F	527.5	0	28.46547	82	49	2	0	2	lens	lens	n	n	POAG	POAG	0	-0.07918	15	15	3	3	-15.68	-21.03
264	42	F	265.5	1		90.33333	70	3	2	5	lens	lens	n	n	POAG	POAG	-0.07918	-0.07918	21	22	4	4	-6.37	-8.24
265	65	M	362	0	20.47827	98.66667	66	3	2	5	lens	lens	n	n	POAG	POAG	0.154902	-0.07918	15	16	3	2	-13.88	-5.04
266	43	M	327	1		78	72	2	2	4	lens	lens	n	n	other	other	0.30103	0.09691	15	14	1	1	-5.68	-4.53
267	76	F	298	0	26.81828	113.6667	68	3	2	5	lens	lens	n	n	other	other	0.045757	0.09691	18	22	4	4	-1.12	-1.66
268	79	M	395.5	0	21.44595	92	56	3	2	5	lens	lens	n	n	POAG	POAG	0.09691	0.09691	13	15	3	3	-8.4	-12.1
269	69	F	334.5	0	18.6058	88.66667	74	3	0	3	lens	lens	n	n	POAG	POAG	0	0.154902	19	18	0	0	-4.67	-0.58
270	72	F	280.5	0	20.52211	98.66667	65	3	2	5	IOL	IOL	n	n	other	other	-0.07918	-0.07918	21	21	2	2	-14.9	-4.66
271	60	M	249	0	23.11712	118	81	3	2	5	lens	IOL	n	n	POAG	POAG	0.30103	-0.07918	28	21	2	2	-16.26	-6.38
272	86	M	166.5	0	21.53791	120.6667	55	2	0	2	IOL	IOL	y	y	EXG	EXG	0.39794	1.39794	23	25	3	3	-18.25	-23.87
274	67	M	175	0	27.4995	109.6667	92	3	2	5	IOL	IOL	n	n	POAG	POAG	-0.07918	-0.07918	31	28	1	1	-12.75	-7.17
275	53	M	122	0	27.78309	115	112	3	2	5	IOL	IOL	n	n	POAG	POAG	-0.07918	-0.07918	21	22	1	1	-17.12	-15.05
276	73	F	288.5	0	27.30095	123.3333	73	3	2	5	IOL	IOL	n	n	other	other	0.045757	-0.07918	20	22	2	2	-2.12	-10.6
277	86	F	501.5	0	23.51111	101.3333	88	2	2	4	IOL	IOL	n	n	other	other	0.045757	-0.07918	25	24	1	1	-10.23	-11.86
278	50	M	428.5	0	18.35838	91.66667	83	3	2	5	IOL	IOL	n	n	POAG	POAG	-0.07918	-0.07918	23	22	4	4	-9.59	-3.77
282	73	M	151.5	0	25.5307	98.33333	75	2	2	4	IOL	IOL	n	n	POAG	POAG	0	-0.07918	21	21	4	4	-15.59	-15.85
283	81	M	172	0	19.01611	102	67	2	2	4	lens	IOL	n	n	other	other	-0.07918	2.69897	17	37	2	3	-6.21	-30.32
284	69	M	123.5	0	27.30311	107	85	3	2	5	IOL	IOL	n	n	POAG	POAG	0.09691	-0.07918	22	22	3	3	-11.57	-22.59
290	77	F	537	0	22.53906	118.6667	85	3	2	5	IOL	IOL	n	n	POAG	POAG	0.221849	0.30103	20	21	2	2	-19.54	-15.54
291	57	F	265.5	0	33.62209	99.66667	86	3	2	5	lens	lens	n	n	POAG	POAG	-0.07918	0.30103	17	15	3	3	-2.65	-13.54
292	87	F	291	0	25.63117	85	77	1	2	3	IOL	IOL	y	y	EXG	EXG	0.154902	2.60206	25	23	1	1	-17.15	-30.29
293	71	M	243	0	22.83737	102.3333	75	3	2	5	IOL	IOL	n	n	POAG	POAG	0.39794	0.154902	44	22	3	3	-15.48	-11.15
294	79	M	202	0	23.33768	138	69	2	2	4	IOL	lens	y	n	EXG	other	0.522879	0.522879	27	20	3	2	-21.58	-7.76
295	64	M	394.5	0	20.19509	93	61	3	2	5	lens	lens	n	n	POAG	POAG	0	0.30103	25	27	4	4	-15.85	-30.23
297	82	F	373.5	0	18.54935	101.3333	72	2	2	4	IOL	IOL	y	y	EXG	EXG	0.39794	0.39794	17	21	3	3	-4.22	-7.31
298	84	F	356.5	0	20.66116	95	117	3	2	5	IOL	IOL	y	y	EXG	EXG	0.823909	0.69897	15	21	0	4		
301	74	M	427	0	23.71184	101.6667	72	3	2	5	IOL	lens	n	n	POAG	other	-0.07918	0	31	24	2	2	-6	-0.27
304	62	F	182.5	0	32.04998	115.3333	75	3	2	5	IOL	IOL	n	n	POAG	POAG	0.522879	0	25	17	0	1	-20.18	-17.57
310	35	M	170	1	21.2585	91.33333	70	1	2	3	lens	lens	n	n	POAG	POAG	-0.07918	0.154902	52	57	4	4	-3.37	-25.47
312	77	M	325	0	20.86112	115.3333	70	3	2	5	IOL	IOL	n	n	POAG	POAG	0.221849	-0.07918	13	24	2	2	-11.27	-15.28
313	59	M	306	0	22.49135	102.3333	82	3	2	5	lens	lens	n	n	POAG	POAG	0	0.09691	15	15	4	4	-10.31	-9.65
314	61	F	405.5	0	18.7215	85	75	3	2	5	IOL	IOL	n	n	POAG	POAG	-0.07918	-0.07918	18	19	3	3	-2.22	-4.03
315	64	M	525	0	22.40879	131	67	3	0	3	lens	lens	n	n	POAG	POAG	0.221849	-0.07918	17	17	3	3	-6.48	-8.92
316	64	M	410.5	0	21.20311	94.66667	70	3	2	5	lens	lens	n	n	POAG	POAG	-0.07918	-0.07918	16	16	4	4	-10.99	-5.42
320	64	F	460	0	20.02884	99.33333	74	3	2	5	lens	lens	n	n	POAG	POAG	0	0.221849	19	23	1	1	-1.77	-23.27
321	73	F	300.5	0	37.46098	127.6667	80	3	2	5	IOL	IOL	n	n	other	other	-0.07918	0.045757	34	59	5	5	-3.24	-4.97
322	38	M	400	0	19.36983	87	83	3	2	5	lens	lens	n	n	other	other	-0.07918	-0.07918	16	9	4	1	-0.9	-2.53
323	64	F	402	0	22.39481	87	62	3	2	5	lens	lens	n	n	POAG	POAG	0.30103	0.154902	20	20	4	4	-10.97	-13.76
324	86	M	385.5	0	21.41094	108	83	1	2	3	lens	lens	y	y	EXG	EXG	0.823909	0.221849	21	23	4	4	-11.06	-9.53
326	51	F	323	0	24.45606	87	63	3	2	5	IOL	IOL	n	n	POAG	POAG	0.522879	0.045757	18	18	3	3	-24.65	-15.24
333	75	F	369.5	0	25.77831	94	68	2	2	4	IOL	IOL	n	y	other	EXG	1.221849	0	13	11	0	3		-5.77
334	87	M	225.5	0	22.89282	101.6667	86	0	0	0	IOL	IOL	n	n	POAG	POAG	-0.07918	0.522879	13	13	0	0	-29.17	-29.61
341	76	M	532.5	0	24.43519	104.3333	80	2	2	4	IOL	IOL	n	y	POAG	EXG	0.09691	0.221849	21	28	3	0	-2.77	-24.87
342	84	M	354.5	0	24.21875	107.6667	64	3	0	3	IOL	IOL	n	n	POAG	POAG	0.09691	0	14	16	3	3	-31.45	-27.69
343	78	F	341.5	0	23.5516	104.6667	72	1	2	3	lens	lens	n	n	other	other	0.39794	0.823909	13	13	4	4	-16.13	-33.3
344	55	M	403			95	72	3	2	5	lens	lens	n	n	POAG	POAG	0.69897	0	12	12	2	2	-12.17	-21.48
345	87	F	268	0	28.30533	112	75	2	0	2	IOL	IOL	y	n	EXG	POAG	0.39794	0.045757	30	24	4	4	-30.29	-10.78
346	65	M	368	0	22.89282	89	78	3	2	5	lens	lens	n	n	POAG	POAG	0.045757	-0.07918	23	25	3	3	-5.13	-9.63
347	70	F	392	0	16.89189	99.33333	62	3	2	5	lens	lens	n	n	POAG	POAG	-0.07918	-0.07918	17	18	3	3		
348	73	M	246	0	24.50895	111.3333	67	3	2	5	IOL	IOL	n	n	POAG	POAG	0.522879	0.154902	31	46	3	3	-20.11	-19
352	72	M	78	0	19.60519	113.6667	84	3	2	5	lens	lens	n	n	other	other	0.39794	0.522879	25	25	3	3	-5.58	-4.56
353	65	F	380.5	0	23.92569	52		3	2	5	IOL	IOL	n	n	other	POAG	0.045757	-0.07918	21	24	3	3	-13.08	-4.45
362	69	F	265.5	0	20.17264	89.33333	66	3	2	5	IOL	IOL	n	n	POAG	POAG	0.09691	1.221849	22	38	3	3	-26.45	-28.7
363	63	F	432	0	20.88889	110	64	3	2	5	IOL	IOL	n	n	POAG	POAG	0.39794	0.09691	26	26	3	3	-9.59	-3.92
367	83	M	197.5	0	24.09297	95	92	3	2	5	IOL	IOL	y	y	EXG	EXG	0.30103	-0.07918	19	17	1	1	-27.44	1.31
372	43	M	191.5	1	25.61728	130.3333	77	2	2	4	lens	lens	n	n	other	other	0	-0.07918	13	14	4	4	-24.29	-25.15
373	81	F	208.5	0	29.5858	71	66	0	0	0	IOL	IOL	n	n	POAG	POAG	-0.07918	0.045757	14	12	1	1	-27.06	-30.67
374	84	M	146	0	21.48438	105.6667	74	3	2	5	lens	lens	n	n	other	other	0.39794	0	24	19	2	0	-4.5	0.45
375	78	M	172.5	0	18.46764	100	63	3	2	5	IOL	lens	n	n	other	other	0.39794	0.045757	15	14	4	3	-26.6	-1.47
376	88	M	450	0	22.21368	110	102	3	2	5	IOL	IOL	y	y	EXG	EXG	1.522879	-0.07918	43	25	3	1	-26.82	-5.33

377	60	M	493.5	1	26.34649	118.3333	64	3	2	5	lens	lens	n	n	POAG	POAG	-0.07918	0	14	14	3	3	-18.84	-18.85
378	78	M	365	1	17.91509	124.3333	59	3	2	5	lens	IOL	y	y	EXG	EXG	0	0.221849	27	25	4	4	-18.48	-17.04
379	76	F	240.5	1	20.11971	113	68	3	2	5	lens	lens	n	y	other	EXG	0	0.221849	17	15	0	3	-4.06	-13.67
383	74	M	361.5	0	19.57168	104.6667	81	3	2	5	IOL	IOL	n	n	POAG	POAG	0.045757	0	25	28	3	3	-11.19	-12.17
385	73	M	533	0	21.15529	104.3333	63	3	2	5	lens	IOL	n	y	POAG	EXG	-0.07918	0	17	29	3	3	-11.28	-11.86
387	66	M	191	0	21.2585	93.66667	79	3	2	5	lens	lens	n	n	POAG	POAG	-0.07918	0.522879	23	27	2	0	-27.8	-31.59
389	81	F	241.5	0	26.37024	99	60	0	2	2	IOL	IOL	y	n	EXG	POAG	-0.07918	-0.07918	45	33	2	2	-13.35	0.25
390	66	M	260.5	1	17.84652	98.66667	105	3	2	5	IOL	IOL	n	n	POAG	POAG	2.69897	1.221849	54	27	3	3		
391	79	M	530.5	0	20.70313	76.33333	62	3	2	5	IOL	IOL	y	y	EXG	EXG	0	2.60206	25	36	3	3	-13.95	-33.52
392	81	F	433.5	0	18.51081	105.3333	103	0	0	0	lens	IOL	n	n	POAG	POAG	0.69897	1.69897	10	12	0	0		
394	69	M	295.5	0	22.15102	90.33333	57	3	2	5	IOL	IOL	y	y	EXG	EXG	-0.07918	-0.07918	21	21	2	2	-14.57	-0.9
396	60	F	391.5	0	19.70553	95	75	3	2	5	lens	lens	y	n	other	other	2	0	19	18	3	3	-27.97	-21.77
403	52	F	553	0	20.96436	79.33333	62	3	2	5	lens	lens	n	n	POAG	POAG	0.823909	0.522879	20	20	4	0	-12.22	-6.26
404	86	M	157.5	0	25.25952	114	83	3	2	5	lens	lens	n	n	POAG	POAG	0.522879	0.221849	13	14	1	1	-12.71	-18.97
405	80	F	382.5	0	21.42857	132.3333	68	3	2	5	IOL	IOL	n	n	POAG	POAG	0	-0.07918	14	15	3	3	-1.76	-13.33
406	78	M	463	0	25	92.33333	74	3	2	5	lens	lens	n	n	POAG	POAG	0.221849	0.30103	15	11	1	0	-2.32	-0.7
407	61	F	282	0	30.46875	105.6667	68	3	0	3	IOL	lens	n	n	POAG	POAG	2.886057	0.69897	8	23	1	5		
411	48	M	169.5	0	21.0772	113.6667	97	3	2	5	IOL	IOL	y	n	EXG	other	0.154902	-0.07918	29	21	2	0	-10.89	-0.02
419	59	F	319.5	0	16.609	94	71	3	2	5	IOL	IOL	n	n	POAG	POAG	-0.07918	-0.07918	15	13	2	2	-10.09	-1.59
420	71	M	368	0	28.22839	108.3333	101	2	2	4	lens	IOL	n	n	POAG	POAG	0.221849	0.154902	9	14	1	1	-2.52	-5.07
423	67	M	184	0	21.51386	112	85	1	2	3	IOL	IOL	n	n	other	other	0.39794	0.045757	12	13	3	1	-30.24	-6.16
424	58	M	302.5	0	23.79536	119	68	2	2	4	lens	lens	n	n	POAG	POAG	0	-0.07918	47	23	4	4	-2.06	0.81
426	61	F	229.5	0	20.96436	97.33333	72	3	2	5	lens	lens	n	n	POAG	POAG	0	0.823909	18	18	4	4	-26.05	-31.07
428	86	F	186	0	23.01118	115.3333	79	2	0	2	IOL	IOL	n	y	POAG	EXG	0.154902	0.522879	19	34	1	1	-12.83	-20.96
429	67	M	234	1	22.30815	104.3333	79	2	0	2	IOL	IOL	y	n	EXG	POAG	0.221849	0	19	12	2	2	-20.42	-4.35
431	68	F	247.5	0	22.95909	108	92	2	2	4	IOL	IOL	n	n	POAG	POAG	-0.07918	-0.07918	26	28	2	2	-11.48	-9.41
439	76	F	501.5	0	17.6693	106.6667	72	3	2	5	IOL	lens	n	n	other	other	0.09691	0.30103	15	21	3	3	-15.32	-7.42
440	69	M	360.5	0	23.87543	115	91	2	2	4	IOL	IOL	n	n	POAG	POAG	0.522879	-0.07918	17	17	2	3	-28.76	-10.68
441	74	M	590	0	21.00767	105.3333	70	2	2	4	lens	IOL	n	n	POAG	POAG	0	0.09691	19	27	4	0	-20.09	-23.97
444	74	M	268.5	0	22.10029	112	77	3	2	5	IOL	IOL	y	n	EXG	other	0.09691	-0.07918	25	17	3	1	-21.94	-2.31
445	85	F	347	0	18.2615	93	73	3	2	5	lens	lens	y	y	EXG	EXG	0.221849	0.39794	13	19	1	1	-2.08	-4.97
446	55	F	226.5	0	29.96878	105.3333	93	3	2	5	lens	lens	n	n	other	other	1.39794	0	13	30	0	4	-32.18	-15.08
447	89	F	478.5	0	25.22409	105.6667	52	2	2	4	IOL	IOL	y	y	EXG	EXG	0.221849	0.045757	26	23	4	4	-29.78	-14.64
448	85	F	242.5	1	22.76147	99.66667	96	1	2	3	IOL	IOL	n	n	other	other	-0.07918	-0.07918	18	24	2	2	-2.5	-4.48
451	47	M	218.5	0	21.29529	105.3333	60	3	2	5	lens	lens	n	n	other	other	0.39794	-0.07918	17	17	3	3	-18.46	-17.94
454	73	M	350	0	25.60554	114.6667	64	2	2	4	lens	lens	n	n	POAG	POAG	0.154902	-0.07918	21	18	0	3	-25.68	-4.12
455	65	F	433	0	25.33333	95.66667	70	3	2	5	IOL	IOL	n	n	other	other	0.09691	-0.07918	63	19	2	1	-21.84	-1.49
463	61	F	186.5	0	18.75	120	65	2	2	4	lens	lens	n	n	POAG	POAG	0.30103	-0.07918	21	19	2	2	-6.03	-5.52
464	70	M	290	0	21.38594	140.3333	75	3	2	5	IOL	IOL	y	n	EXG	POAG	0.09691	-0.07918	25	19	4	4	-26.02	-0.01
465	75	M	290.5	0	22.77319	76.66667	62	3	2	5	lens	lens	n	n	POAG	other	0	0	32	15	2	2	-0.51	1.68
466	69	M	302.5	0	22.03857	100.3333	65	2	2	4	lens	lens	n	n	POAG	POAG	0	-0.07918	15	17	4	4	-15.52	-10.95
468	45	M	220.5	0	20.19947	117.6667	67	3	2	5	lens	lens	n	n	POAG	POAG	-0.07918	-0.07918	18	13	0	1	-3.67	-11.31
473	83	F	272.5	0	21.91381	77.33333	87	2	2	4	IOL	IOL	y	y	EXG	EXG	2	0.522879	15	13	4	4	-29.18	-15.31
478	72	F	268.5	0	23.30905	97	80	2	2	4	IOL	IOL	n	n	POAG	POAG	0.09691	1.69897	15	15	2	0	-14.28	
481	59	M	286	0	21.36687	115	72	2	2	4	lens	lens	n	n	POAG	POAG	2.60206	0.30103	23	27	3	3	-25.14	-27.27
483	67	F	369	0	27.63037	106.6667	82	2	2	4	lens	lens	n	n	POAG	POAG	-0.07918	-0.07918	29	23	3	3	-1.24	-2.22
484	83	M	334.5	0	23.42356	90.33333	67	1	2	3	IOL	IOL	y	n	EXG	POAG	0.39794	0.045757	18	14	4	0	-4.51	-2.08
485	72	M	342	0	22.75831	107.6667	77	3	2	5	IOL	IOL	n	n	POAG	POAG	0.045757	0.30103	24	23	4	4	-12.09	-22.76
487	71	F	524	0	24	116	54	3	2	5	lens	lens	n	n	POAG	POAG	-0.07918	-0.07918	22	27	4	4	-4.66	-9.12
488	80	F	460.5	0	27.20961	111.3333	62	3	2	5	lens	lens	n	n	POAG	POAG	0.39794	0.39794	13	11	3	3	-5.83	-18.22
489	86	M	444.5	0	21.77844	94	76	1	2	3	IOL	IOL	y	y	EXG	EXG	0.39794	0.09691	49	27	3	2	-21.66	-2.18
491	66	F	263	0	23.93606	102.6667	67	3	2	5	IOL	IOL	n	n	POAG	POAG	0.69897	0.154902	19	61	2	2	-29.57	-15.52
495	65	F	319.5	0	28.69265	111.6667	71	3	2	5	lens	lens	n	y	other	EXG	-0.07918	-0.07918	19	31	0	2	0.07	-15.77
507	68	F	369.5	0	21.50447	80	56	3	2	5	lens	lens	n	n	POAG	POAG	-0.07918	0.154902	13	13	4	4	0.99	-19.32
508	65	M	409.5	0	26.06168	0		2	2	4	lens	lens	n	n	POAG	POAG	-0.07918	0	14	14	3	3	-10.53	-16.73
509	60	F	363.5	1	18.82711	84	98	3	2	5	lens	lens	n	n	other	other	0.09691	0.045757	17	17	1	1	-3.71	-14.67
512	78	F	254.5	0	23.14726	87.66667	64	2	2	4	IOL	IOL	n	n	POAG	POAG	0.154902	0	12	10	3	3	-25.3	-8.52
514	78	F	736	0	18.46764	77.33333	61	3	2	5	IOL	IOL	n	n	other	other	0	0.30103	16	37	2	2	-2.45	-24.62
515	52	M	348	0	20.3125	86.33333	75	3	2	5	IOL	IOL	n	n	POAG	POAG	0.221849	0.39794	15	17	2	3	-7.96	-19.5
516	69	M	313.5	0	23.14726	79.33333	57	3	2	5	lens	IOL	y	n	EXG	other	0.154902	-0.07918	17	17	4	0	-11.01	0
518	63	M	213.5	0	26.33289	124.3333	73	3	2	5	lens	IOL	n	y	other	EXG	-0.07918	0	30	30	3	3	1.13	-0.5
519	59	M	355	1	26.77551	107.3333	84	3	2	5	IOL	IOL	n	n	POAG	POAG	-0.07918	0.09691	20	25	3	3	-8.74	-19.23
520	75	F	391	0	25.07716	99	71	3	2	5	lens	IOL	y	y	EXG	EXG	-0.07918	1.69897	23	29	2	2	-0.96	
529	67	M	285	1	26.92744	112.6667	72	3	0	3	IOL	lens	y	n	EXG	other	0.045757	0	27	21	2	0	-1.78	-2.14
530	48	F	449.5	0	19.10009	70	57	3	0	3	lens	lens	n	n	POAG	POAG	-0.07918	0.39794	22	21	4	4	-17.97	-17.53
531	64	M	291.5	0	20.42942	96.66667	75	2	0	2	IOL	IOL	y	y	EXG	EXG	0	0.09691	19	37	3	3	-5.32	-28.26
532	77	M	447.5	0	22.77319	80	62	3	2	5	IOL	IOL	n	n	POAG	POAG	0.09691	-0.07918	20	20	3	3	-24.92	-15.98
533	64	M	242.5	0	23.72281	110.6667	69	3	2	5	IOL	IOL	n	n	POAG	POAG	-0.07918	-0.07918	21	19	3	3	-20.14	-11.75

534	65	M	220	1	21.63115	106.6667	73	2	2	4	IOL	IOL	n	n	POAG	POAG	0	-0.07918	18	21	1	1	-10.84	-16.89
539	73	M	213	0	20.54989	97.66667	56	3	2	5	lens	lens	n	n	other	other	0.221849	0.154902	20	17	4	4	-24.71	-0.95
540	71	M	538	1	23.04688	110.3333	69	2	2	4	lens	lens	y	y	EXG	EXG	0	-0.07918	18	23	5	5	-0.37	-13.76
545	89	F	250	0	20.44444	91	94	3	0	3	IOL	IOL	y	n	EXG	other	0.522879	2	37	18	2	2		
546	42	M	277	0	22.14533	96.33333	84	3	2	5	lens	lens	n	n	other	other	0.09691	1.30103	20	20	4	4	-20.32	-21.01
548	70	M	186	0	19.15119	86.66667	65	2	2	4	lens	lens	n	n	POAG	POAG	-0.07918	-0.07918	19	20	4	4	-9.39	1.21
549	63	M	146.5	1	20.32225	89.33333	83	2	2	4	IOL	IOL	n	n	POAG	POAG	0.045757	0.39794	19	23	4	4	-20.73	-19.61
550	68	M	196	1	23.99946	124.6667	73	3	2	5	lens	lens	n	y	other	EXG	-0.07918	-0.07918	21	29	2	0	0.51	-1.02
552	60	M	338.5	1	21.73651	118.3333	95	2	2	4	IOL	IOL	n	n	other	other	0.045757	-0.07918	30	21	2	0	-7.41	-2.81
561	81	F	316.5	0	27.7671	100.6667	79	2	0	2	IOL	IOL	y	y	EXG	EXG	0.39794	0.09691	14	25	2	2	-28.9	-18.28
562	77	M	364.5	0	27.68878	101	64	3	2	5	IOL	IOL	n	n	POAG	POAG	0	-0.07918	21	21	3	3	-15.34	-10.76
566	90	M	261	0	23.06805	84.66667	77	0	2	2	IOL	IOL	n	y	other	EXG	0.154902	0.39794	24	40	3	3	-10.34	-30.21
568	64	M	190.5	0	31.77393	118.6667	76	3	2	5	IOL	IOL	n	n	other	other	-0.07918	-0.07918	21	32	5	5	-14.23	-9.58
571	71	M	342	1	20.33833	104.3333	80	3	2	5	lens	lens	n	n	other	other	2.69897	0	15	17	3	3	-30.24	-29.06
580	67	F	571.5	0	25.10957	107.6667	70	3	2	5	lens	lens	n	n	other	other	0.39794	0.045757	34	37	4	4	-24.23	-15.51
584	60	M	530	0	23.87511	118.3333	66	3	2	5	IOL	IOL	y	n	POAG	POAG	-0.07918	-0.07918	22	18	4	4	-24.21	-17.41
586	72	F	318.5	0	25.20809	110.3333	67	3	2	5	IOL	IOL	n	n	POAG	POAG	0.154902	-0.07918	17	18	4	4	-25.1	-19.7
588	76	M	241	0	25.34435	108	74	3	2	5	IOL	IOL	n	n	POAG	POAG	0.045757	0.221849	14	16	3	3	-3.44	-18.94
589	66	F	417.5	0	21.48438	87	76	1	2	3	lens	lens	n	n	POAG	POAG	0.522879	1	34	37	4	4	-20.02	-19.27
590	67	M	204	0	17.44126	82.66667	101	0	2	2	IOL	IOL	n	n	POAG	POAG	0	0.09691	21	21	3	3	-12.93	-13.51
595	62	M	300.5	0	22.85714	102.3333	52	3	2	5	IOL	lens	n	n	POAG	POAG	1	-0.07918	15	16	4	4	-33.89	-17.04
596	41	F	290	0	19.38214	86.66667	73	3	2	5	lens	lens	n	n	POAG	POAG	-0.07918	-0.07918	45	50	0	0	-3.87	-2.07
597	61	M	210	1	28.69898	104	82	3	2	5	IOL	IOL	n	n	POAG	POAG	0.39794	0.522879	30	21	4	4	-31.14	-9.93
598	75	F	307.5	0	20.83	111	71	2	2	4	IOL	IOL	y	y	EXG	EXG	0.045757	0.09691	25	17	4	4		
599	84	F	347	0	26.02041	83.33333	76	2	0	2	IOL	IOL	n	n	POAG	POAG	0.045757	0	25	17	2	2	-8.66	-4.52
602	71	M	236.5	1	21.97134	81.66667	70	3	2	5	lens	lens	n	n	POAG	POAG	-0.07918	-0.07918	19	18	2	2	-2.45	-19.97
603	77	M	573	0	21.51386	83.33333	80	3	2	5	lens	IOL	n	n	POAG	POAG	0.09691	0	19	29	4	4	-0.1	-18.5
606	82	F	290.5	0	18.01721	91.33333	72	3	2	5	IOL	IOL	y	y	EXG	EXG	0.154902	0.39794	17	44	0	0	-10.3	-28.74
608	61	M	378.5	0	23.78121	104.6667	62	3	2	5	IOL	IOL	n	n	POAG	POAG	-0.07918	0	17	17	4	2	-23.85	-23.58
609	48	F	206	1	22.4323	103.6667	97	3	2	5	lens	lens	n	n	POAG	POAG	0	0	21	21	3	3	-0.99	-6.08
611	67	M	257	0	25.05931	110.6667	78	3	2	5	IOL	IOL	n	n	POAG	POAG	-0.07918	0.09691	18	19	3	3	-15.08	-19.73
612	71	M	349.5	0	21.67126	94.66667	77	2	2	4	IOL	IOL	n	n	POAG	POAG	0.09691	0	25	34	0	4	-0.4	-7.07
618	86	F	203.5	0	26.22222	132.6667	72	1	0	1	lens	IOL	n	n	POAG	POAG	2.79588	1.522879	15	9	3	2		
619	77	F	115.5	0	27.34375	101	93	3	2	5	lens	IOL	n	n	POAG	POAG	0.154902	0.09691	11	13	2	2	-4.84	-6.65
620	83	M	316.5	0	22.49135	102.3333	70	1	2	3	lens	lens	n	y	POAG	EXG	0.09691	0.30103	9	14	5	5	-0.77	-17.32
621	82	M	168	0	26.83865	98.33333	79	3	2	5	lens	IOL	n	y	other	EXG	0.221849	0.30103	15	21	0	4	-0.22	-29.91
622	77	F	283	0	20.8292	86	80	3	2	5	lens	lens	n	n	POAG	POAG	0.09691	0	11	12	2	2	-2.01	-7.1
624	52	M	292	0	21.71807	99	64	3	2	5	lens	lens	n	n	POAG	POAG	0	0	15	15	4	4	-20.73	-24.1
625	58	M	181.5	0	23.87511	112.3333	89	3	2	5	lens	lens	n	n	POAG	POAG	-0.07918	-0.07918	21	18	4	4	-3.03	-12.76
631	50	M	428.5	0	18.83239	100.6667	78	3	2	5	lens	lens	n	n	POAG	POAG	-0.07918	-0.07918	23	22	4	4	-9.59	-3.77
632	84	M	132	0	26.67276	99.33333	72	2	2	4	IOL	IOL	n	n	POAG	POAG	0.221849	0.221849	14	15	3	3	-11.25	-23.57
633	55	F	340	0	24.03441	103.3333	69	3	2	5	lens	lens	n	n	POAG	POAG	0.09691	-0.07918	19	25	1	1	-18.25	-9.02
634	78	F	671	0	25.29938	119.6667	68	3	2	5	IOL	IOL	n	n	POAG	POAG	-0.07918	-0.07918	18	17	3	3	-3.44	-10.16
645	57	M	230.5	1	19.83516	100	77	3	2	5	lens	lens	n	n	POAG	POAG	-0.07918	-0.07918	19	17	4	4	-22.49	-21.43
646	81	M	481	0	23.05175	102.6667	81	3	2	5	IOL	IOL	n	n	POAG	POAG	0.045757	0.09691	18	18	0	0	-6.04	-9.73
653	47	F	174	0	23.13851	94	82	3	2	5	lens	lens	n	n	other	other	-0.07918	1.045757	16	13	4	4	-2.1	-22.28
654	59	M	170	0	27.60945	0		3	2	5	lens	lens	n	n	other	other	-0.07918	0.154902	19	21	4	0	-0.1	-19.47
666	73	F	338	0	19.43635	115.3333	77	3	2	5	lens	lens	n	y	POAG	EXG	0.221849	0.39794	16	16	1	4		
667	85	M	85	0	21.79931	102.3333	76	2	2	4	IOL	lens	y	n	EXG	other	1.154902	0.154902	23	19	3	3		-19.2
669	67	M	273	1	24.4898	117.3333	60	3	2	5	IOL	IOL	n	n	POAG	POAG	0.09691	-0.07918	15	15	2	2	-5.42	-2.8
672	85	M	220.5	0	21.875	104	78	2	2	4	IOL	IOL	y	y	EXG	EXG	-0.07918	2	13	35	0	2	-1.31	-30.47
673	80	F	554	0	21.58003	90	67	3	2	5	IOL	IOL	y	n	EXG	POAG	0.045757	-0.07918	27	21	1	1	-8.13	-12.24
674	65	F	355.5	0	29.31986	90.66667	64	3	2	5	IOL	IOL	y	y	POAG	POAG	0	0.154902	40	19	0	4	-15.24	-6.69
681	92	F	183.5	0	17.5981	78	72	3	2	5	IOL	IOL	y	y	EXG	EXG	0.30103	-0.07918	27	17	4	4	-28.64	-0.37
682	64	M	281.5	0	23.99946	106.6667	65	2	2	4	IOL	lens	y	n	EXG	other	0.522879	0	25	19	2	0	-22.35	-2.83
689	47	M	608.5	0	36.98192	113	99	3	2	5	lens	lens	n	n	other	other	0.69897	0.221849	32	31	3	3	-25.47	-22.65
690	69	M	147	1	21.38594	75.33333	53	3	2	5	IOL	IOL	n	n	POAG	POAG	0	-0.07918	21	19	4	4	-29.84	-20.43
693	81	F	497	0	26.84067	80.66667	69	3	2	5	IOL	IOL	n	n	POAG	POAG	-0.07918	0.045757	16	16	2	2	-2.29	-10.51
695	85	F	445.5	0	23.73996	87.66667	63	2	2	4	lens	lens	n	n	POAG	POAG	0.39794	0.39794	12	13	1	1		
696	81	M	166.5	0	22.67574	103.6667	54	0	2	2	IOL	IOL	n	n	POAG	POAG	0.09691	0	17	20	3	3	-6.62	-15.01
697	66	M	139	0	19.95728	0		3	2	5	IOL	IOL	n	n	POAG	POAG	-0.07918	0	20	22	4	4	-19.26	-18.52
702	73	F	372.5	0	25.77778	85.66667	86	3	2	5	lens	lens	n	n	POAG	POAG	-0.07918	0	19	19	2	2	-0.65	-1.08
703	71	F	334.5	0	23.68317	107	99	3	2	5	IOL	IOL	n	n	POAG	POAG	0.045757	-0.07918	19	19	1	1	-10.62	-1.54
706	50	M	210	0	22.79036	109.6667	57	3	2	5	lens	lens	n	n	other	other	1.045757	-0.07918	29	17	4	4		-1.01
719	71	M	484	0	18.25311	97.66667	61	3	2	5	lens	lens	n	n	POAG	POAG	-0.07918	-0.07918	21	21	1	1	-3	-0.38
720	50	F	284	0	23.833	90.33333	96	3	2	5	lens	lens	n	n	other	other	-0.07918	2.60206	14	17	0	0	0	-18.87
730	61	M	258.5	0	26.5625	148.3333	87	3	2	5	lens	IOL	n	y	other	EXG	2.886057	0.09691	69	33	0	2		-20.91
735	79	M	803.5	0	22.86253	94.66667	60	2	2	4	lens	lens	n	n	other	other	0.39794	0.39794	13	15	2	2	-7.46	-12.04

739	72	M	312.5	0	26.02617	118	64	3	2	5	IOL	IOL	n	n	POAG	POAG	0.522879	0.221849	15	16	1	0	-24.34	-27.38
740	64	M	287	0	23.25502	102.6667	75	3	2	5	lens	lens	y	y	EXG	EXG	-0.07918	-0.07918	27	21	0	0	-2.37	-0.57
742	80	M	367.5	0	24.4646	112.6667	55	3	2	5	lens	lens	n	n	other	other	0.30103	0.045757	28	14	4	1	-28.68	-2.11
743	79	M	349	0	22.65625	100.3333	65	2	2	4	IOL	lens	n	n	POAG	POAG	0.522879	0.39794	19	18	4	4	-21.95	-13.65
758	69	F	330	0	19.47715	90	80	3	2	5	IOL	IOL	y	y	EXG	EXG	-0.07918	0.09691	20	17	3	3	-12.92	-12.22
772	76	M	291.5	0	24.34176	78	74	2	0	2	lens	IOL	n	y	POAG	EXG	0.39794	0.09691	22	59	3	3	-2.18	-25.64
775	67	M	157.5	1	25.14861	113.6667	56	3	2	5	IOL	lens	n	n	POAG	POAG	0.045757	-0.07918	10	15	4	4	-26.68	-10.28
778	49	M	258.5	1	22.13674	101.6667	68	3	2	5	lens	lens	y	n	EXG	other	0.045757	-0.07918	40	15	2	1	-2.83	-3.18
793	83	F	391.5	0	25.45236	97	99	3	2	5	lens	lens	y	y	other	other	2.69897	0.154902	55	14	0	0		
796	81	F	448	0	23.73996	94	63	2	2	4	IOL	IOL	n	y	POAG	EXG	-0.07918	-0.07918	20	20	3	3	-9.73	-19.28
816	74	M	278	0	22.94213	0		3	2	5	IOL	IOL	n	n	POAG	POAG	0.522879	0.522879	18	19	5	5	-30.22	-25.46
818	80	F	449	0	26.74048	85	68	2	2	4	IOL	IOL	n	n	POAG	POAG	0.39794	0	24	25	2	2	-24.87	-8.13
819	51	M	194.5	1	26.02617	108	60	3	2	5	lens	lens	n	n	POAG	POAG	-0.07918	-0.07918	21	18	5	5	-10.75	-13.64
821	76	M	103.5	0	19.94806	122.3333	70	2	2	4	IOL	IOL	y	y	other	other	0.30103	1.045757	28	24	3	3	-21.36	-24.81
831	82	F	315	0	28.62147	91	68	2	2	4	IOL	IOL	n	n	POAG	POAG	-0.07918	0	17	17	2	2	-12.01	-2.37
839	77	M	452.5	0	19.78997	119.6667	82	2	2	4	IOL	IOL	n	n	POAG	POAG	0.09691	0.09691	17	19	4	4	-22.3	-12.92
849	67	M	223.5	1	25.01352	109	95	3	2	5	lens	lens	n	n	POAG	POAG	0.221849	0.221849	16	15	2	2	-3.11	-15.06
853	52	F	281	1	24.45606	83	72	3	2	5	lens	lens	n	n	POAG	POAG	-0.07918	-0.07918	17	21	4	4	-8.03	-8.77
854	69	F	415	0	24.23823	91	71	3	2	5	lens	lens	n	n	POAG	POAG	0	0.30103	20	20	4	4	-8.84	-9.73
855	77	M	417	0	23.87511	135.6667	69	2	2	4	lens	lens	y	n	EXG	POAG	1.69897	0.154902	14	11	4	4	-26.98	-10.9
865	67	F	355	0	22.06035	107.6667	68	3	2	5	IOL	IOL	n	n	other	other	0	0.522879	23	22	3	3	-9.89	-18.89
874	86	M	199.5	1	22.75831	97.33333	77	2	2	4	lens	lens	y	y	EXG	EXG	0.69897	0.522879	13	14	0	0	-5.68	-2.28
876	83	F	252.5	0	20	106	94	3	2	5	lens	lens	n	n	other	other	0.522879	2.79588	31	48	0	2	-8.96	-30.53
878	75	F	339.5	0	23.06805	101	66	3	2	5	IOL	IOL	n	n	POAG	POAG	0.09691	-0.07918	19	15	3	3	-0.11	0.83
895	74	M	293	0	25.81663	115.6667	59	3	2	5	IOL	lens	n	n	POAG	other	-0.07918	2.886057	19	71	4	1	-2.63	
902	54	M	367.5	0	20.70082	92.66667	70	3	2	5	lens	lens	n	n	POAG	POAG	0	0.045757	18	18	3	3	-12.51	-14.33
903	51	M	662	0	23.52941	107	74	3	2	5	lens	lens	n	n	POAG	POAG	-0.07918	-0.07918	19	18	4	4	-6.54	-0.81
904	63	M	725	0	23.58833	101.6667	54	2	2	4	lens	lens	n	n	POAG	other	0	-0.07918	14	12	3	0	-10.77	0.03
905	60	M	166.5	0	19.70796	78	112	3	2	5	lens	lens	n	n	other	other	0.39794	0.39794	12	12	2	2	-13.35	-13.37
906	65	M	544	0	22.10029	85	60	3	2	5	lens	lens	n	n	POAG	POAG	-0.07918	-0.07918	13	11	3	3	-1.41	-6.72
907	59	M	233.5	1	26.07897	92.33333	72	3	2	5	lens	lens	n	n	other	other	0	-0.07918	9	9	3	3	-30.94	-15.14
908	77	F	281.5	0	22.07108	101	65	3	2	5	lens	lens	y	y	EXG	EXG	2.60206	0.045757	16	14	4	1	-29.64	-16.83
909	56	F	249	0	22.76944	91.66667	65	3	2	5	lens	lens	n	n	POAG	POAG	-0.07918	0	20	21	4	4	-5.52	-3.3
914	56	M	295	0	24.80159	112.6667	103	2	2	4	lens	lens	n	n	POAG	POAG	0	0.522879	30	31	3	3	-1.69	-24.21
919	73	M	274.5	0	26.17188	114.6667	96	3	2	5	lens	lens	n	n	POAG	POAG	0	0	15	17	4	4	-17.7	-18.61
924	50	F	324	0	20.61313	136.6667	91	3	2	5	lens	lens	n	n	POAG	POAG	0.045757	-0.07918	22	23	0	0	-3.04	-4.43
928	75	M	395.5	0	22.32143	105	56	3	2	5	IOL	IOL	n	n	other	POAG	0	0.045757	13	11	0	3		
933	59	F	352.5	0	20.95717	87	76	2	2	4	lens	lens	n	n	other	other	-0.07918	-0.07918	31	18	0	1	-19.11	-0.86
948	58	M	181.5	0	23.50781	105	73	3	2	5	lens	lens	n	n	POAG	POAG	-0.07918	0	21	21	4	4	-3.03	-12.76
960	58	M	355.5	0	23.42356	96.33333	50	3	2	5	lens	lens	n	n	other	other	1	1	25	27	4	4		
961	58	M	236	1	18.06973	95	81	2	2	4	lens	IOL	n	n	POAG	POAG	-0.07918	-0.07918	14	25	3	4	-3.65	-20.77
962	65	M	241	0	24.02381	81.33333	75	1	0	1	lens	lens	n	n	POAG	POAG	0.522879	0.522879	11	11	3	3	-26.11	-28.31
963	37	F	269	0	24.85907	100	61	3	2	5	lens	lens	n	n	other	other	-0.07918	-0.07918	18	18	5	5	-9.76	-13.1
970	82	F	300.5	0	15.6956	120.3333	94	3	2	5	lens	lens	y	y	EXG	EXG	0.69897	0.69897	23	25	0	0		
974	84	F	205.5	0	17.98167	84.66667	52	2	2	4	IOL	IOL	y	n	EXG	other	0	-0.07918	25	18	3	3	-23.54	-3.57
978	71	F	444	0	20.56881	83.66667	60	3	2	5	lens	lens	n	n	other	other	0.39794	0.30103	14	15	3	3	-2.27	-3.03
980	66	F	208.5	0	26.22268	90	62	3	2	5	lens	lens	n	n	POAG	POAG	0.045757	-0.07918	16	16	3	3		
983	50	M	279.5	0	19.95936	88	86	3	2	5	lens	lens	n	n	POAG	POAG	-0.07918	0.221849	15	17	1	1	-10.11	-10.08
984	72	M	177.5	0	27.92667	0		2	0	2	IOL	lens	n	n	other	other	1.69897	2.69897	23	12	0	0		
985	87	F	288	0	21.93878	95	67	3	2	5	IOL	lens	y	n	EXG	POAG	-0.07918	0.045757	21	20	1	1		
1000	43	F	501.5	0	21.71925	84.66667	76	3	2	5	lens	lens	n	n	POAG	POAG	-0.07918	-0.07918	23	26	4	4	-13.29	-11.6
1007	84	F	299	0	27.76621	73	52	2	2	4	IOL	IOL	n	n	POAG	POAG	1	0.154902	31	32	4	4	-27.74	-16.3
1008	92	M	436	0	19.14672	88.33333	79	3	2	5	IOL	IOL	n	n	other	other	0.221849	0.30103	23	28	4	4	-23.09	-28.93
1010	85	M	427.5	0	24.03461	125	60	2	2	4	IOL	IOL	y	y	EXG	EXG	0.154902	0.69897	10	10	4	4	-16.94	-32.97
1023	69	F	380.5	0	19.11111	101.6667	67	3	2	5	lens	lens	n	n	other	other	0.154902	-0.07918	14	15	0	0	-5.38	-2.27
1028	45	M	332.5	0	21.30682	87	59	3	2	5	lens	lens	n	n	POAG	POAG	-0.07918	-0.07918	11	14	2	2	-10.06	-0.07
1030	66	M	236.5	1	21.88708	102.3333	69	3	2	5	IOL	lens	n	y	other	EXG	-0.07918	0.39794	15	18	3	3	-5.08	-4.56
1032	73	F	226.5	0	24.19649	112.3333	66	3	2	5	lens	lens	n	n	other	other	0.154902	0.045757	13	13	4	4	-12.01	-8.33
1034	78	F	429.5	0	22.71897	101.3333	65	3	2	5	lens	lens	n	n	POAG	POAG	0.221849	0	9	12	2	2	-6.08	-2.55
1042	74	M	248.5	0	23.82813	95.33333	70	1	2	3	lens	lens	n	n	other	other	-0.07918	-0.07918	35	43	0	1	-3.48	-14.4
1049	67	F	276.5	0	23.62445	93	72	2	2	4	IOL	IOL	n	n	other	other	-0.07918	-0.07918	11	10	1	1	-10.73	-4.37
1060	70	F	266.5	0	12.32652	108	107	3	2	5	lens	lens	n	n	POAG	POAG	0.154902	0.30103	18	17	2	2	-7.37	-12.94
1061	80	F	256.5	0	20.62271	102.6667	87	3	2	5	lens	lens	y	y	EXG	EXG	0.39794	0.39794	9	9	1	1	-6.57	-3.96
1062	77	F	292	0	22.76944	94.66667	70	3	2	5	IOL	IOL	y	y	EXG	EXG	0	0.045757	20	21	4	3	-13.29	-14.96
1063	72	M	280	0	25.31545	98	80	2	2	4	IOL	IOL	n	n	POAG	POAG	0	0	31	23	4	4	-8.1	-3.27
1068	69	M	302.5	0	22.03857	97	63	2	2	4	lens	lens	n	n	POAG	POAG	0	-0.07918	15	17	4	4	-15.52	-10.95
1072	71	M	236.5	1	22.57105	90.33333	66	3	2	5	lens	lens	n	n	POAG	POAG	-0.07918	-0.07918	19	22	2	2	-2.45	-19.97
1075	66	M	235	0	19.57168	103.6667	59	3	2	5	lens	IOL	n	y	other	EXG	-0.07918	-0.07918	16	24	0	1	1.84	-0.6

1080	67	M	311.5	1	20.76125	111	88	1	2	3	lens	lens	n	n	other	other	0.30103	0.69897	19	19	0	0	-6.83	-22.63
1081	77	F	400	0	19.81784	0		3	0	3	lens	lens	n	y	POAG	EXG	0.045757	0.09691	14	22	1	2		
1086	60	M	276	0	29.38776	123.6667	63	3	2	5	lens	lens	n	n	POAG	POAG	-0.07918	-0.07918	16	20	3	4	-3.04	-4.17
1091	66	M	200.5	1	30.11099	120	74	3	2	5	IOL	IOL	y	y	EXG	EXG	0.823909	0.522879	30	21	4	4	-9.21	-2.33
1092	68	F	288.5	0	29.13632	99.66667	75	3	2	5	IOL	IOL	n	n	POAG	POAG	0.69897	0	13	14	5	5	-17.28	-14.12
1093	70	M	290	0	20.98399	136.6667	73	3	2	5	IOL	lens	y	n	EXG	POAG	0.09691	-0.07918	25	19	4	4	-26.02	-0.01
1103	68	F	273.5	0	25.21736	89	69	2	2	4	IOL	IOL	n	n	other	other	0	0.39794	17	21	3	3	-10.8	-9.35
1105	73	M	249	0	22.39541	118	61	3	2	5	IOL	IOL	n	n	POAG	POAG	0.221849	0.522879	27	17	2	0	-28.75	-22.27
1112	77	F	312.5	0	17.54309	88.66667	85	3	2	5	lens	lens	n	n	other	other	0.39794	0.30103	16	17	1	1	-0.98	-1.87
1116	74	F	364.5	0	24.44444	80.33333	72	2	2	4	lens	lens	n	n	other	other	0.30103	0.30103	14	14	2	2		
1123	66	M	179	1	25.63692	74.66667	101	3	2	5	IOL	IOL	n	n	other	other	1	0.30103	35	31	5	5		
1124	68	M	339.5	0	19.00391	73.33333	76	3	2	5	lens	lens	n	n	POAG	other	0.30103	0.522879	25	15	4	0	-30.03	1.65
1125	34	F	263.5	1	23.50781	102.6667	65	3	2	5	lens	lens	n	n	POAG	POAG	-0.07918	-0.07918	11	13	4	4	-14.25	-28.44
1126	64	F	562.5	0	19.14672	106.6667	63	2	2	4	lens	lens	n	n	POAG	POAG	0.045757	0.09691	17	18	4	4	-12.31	-5.87
1129	78	F	345.3333	0	18.369	123	78	3	2	5	lens	lens	n	n	POAG	POAG	0	0.30103	17	21	1	4		
1131	69	M	218	0	22.4323	101.6667	72	3	2	5	lens	IOL	n	n	POAG	POAG	0.154902	0.69897	12	15	5	5	-19.37	-20.19
1141	71	M	267	1	27.13141	104.6667	89	3	2	5	IOL	IOL	y	y	EXG	EXG	0.045757	0	29	27	3	4	-26.63	-2.94
1143	61	F	230	0	18.75	106.6667	67	2	2	4	lens	lens	n	n	POAG	POAG	-0.07918	-0.07918	18	19	3	3	-7.16	-8.42
1154	78	M	405	0	25.86451	84.33333	86	3	2	5	lens	lens	y	y	EXG	EXG	0.221849	0.154902	18	18	2	2	-3.66	-3.42
1163	81	F	398.5	0	21.40309	93	55	2	2	4	lens	lens	n	n	POAG	POAG	-0.07918	0.154902	16	19	2	4	-5.01	-24.45
1166	84	M	260.5	0	25.39063	86.66667	80	1	2	3	IOL	IOL	y	n	EXG	POAG	-0.07918	-0.07918	18	17	3	3		
1167	41	M	270.5	0	37.84988	130.3333	78	3	2	5	IOL	IOL	n	n	POAG	POAG	0	0	18	20	4	4	-14.03	-15.09
1200	48	F	449.5	0	19.10009	70.33333	63	3	0	3	lens	lens	n	n	POAG	POAG	-0.07918	0.39794	22	21	4	4	-20.27	-18.63
1201	70	F	449.5	0	26.83865	93	69	3	2	5	lens	lens	n	n	POAG	POAG	-0.07918	-0.07918	17	18	4	4		
1202	83	F	267	0	19.14672	96.33333	80	3	2	5	IOL	IOL	n	n	POAG	POAG	0.221849	0.39794	11	13	5	5	-24.09	-27.36
1204	66	M	253.5	0	21.96712	90	68	3	2	5	IOL	IOL	n	n	POAG	POAG	1.39794	0.09691	21	19	1	1	-28	-27.38
1205	78	F	224.5	0	22.0741	113	69	3	2	5	IOL	IOL	n	n	POAG	POAG	0.30103	0.522879	24	32	4	4	-19.89	-30.36
1206	71	M	320	0	21.92613	100	60	0	2	2	lens	IOL	n	n	POAG	POAG	0.30103	0.154902	25	55	5	5	-25.55	-27.93
1219	72	F	519	0	21.23057	97.66667	60	3	2	5	IOL	IOL	n	n	POAG	POAG	0.30103	0	23	23	4	4	-24.88	-23.94
1220	54	M	341	0	28.71048	107.3333	80	3	2	5	lens	lens	n	n	POAG	POAG	-0.07918	-0.07918	25	28	4	4	-5.95	-20.9
1228	72	M	360	1	25.24934	111.3333	60	3	2	5	IOL	lens	n	n	other	other	0.39794	0.39794	17	16	0	0	-4.95	-3.24
1238	63	M	273.6667	0	25.78125	100.3333	66	3	2	5	lens	lens	n	n	POAG	POAG	0.221849	0.522879	22	23	4	4	-16.15	-16.82
1239	73	M	236.5	0	23.42356	90	81	2	2	4	lens	lens	y	n	EXG	POAG	0.39794	-0.07918	27	19	4	4	-30.45	0.26
1255	64	M	287	0	23.93899	99.66667	64	3	2	5	lens	lens	y	y	EXG	EXG	-0.07918	-0.07918	27	21	0	0	-2.37	-0.57
1256	65	M	368	0	23.49524	91.33333	79	3	2	5	lens	lens	n	n	POAG	POAG	0.045757	-0.07918	23	25	3	3	-5.13	-9.63
1265	57	M	265.5	0	33.62209	78.66667	65	3	2	5	lens	lens	n	n	POAG	POAG	-0.07918	0.30103	17	15	3	3	-2.65	-13.54
1275	67	M	353.5	0	21.45357	115	52	2	2	4	lens	lens	n	n	POAG	POAG	0.221849	1	16	16	3	3	-9.8	-23.34
1276	77	F	275	0	23.4375	120.3333	78	3	2	5	IOL	IOL	n	n	POAG	POAG	-0.07918	-0.07918	19	19	2	2	-3.06	-8.96
1280	68	M	196	0	22.9854	118	55	3	2	5	lens	lens	n	y	other	EXG	-0.07918	-0.07918	21	29	2	0	0.51	-1.02
1285	67	F	357.5	0	19.31295	128.3333	72	3	2	5	IOL	IOL	n	y	POAG	EXG	-0.07918	-0.07918	22	22	2	2	-1.05	-7.56
1293	81	M	354.5	0	21.48438	81.33333	85	1	2	3	lens	lens	n	y	POAG	EXG	0.30103	0.39794	12	15	0	0	-2.69	-11.2
1304	86	F	358.5	0	27.11111	82.33333	89	0	2	2	IOL	IOL	n	n	other	POAG	0.69897	1.30103	27	29	3	3		
1305	83	F	318	0	22.22222	80.66667	62	3	2	5	lens	lens	n	n	POAG	POAG	1.522879	0.69897	13	16	0	0	-16.21	-11.45
1312	74	M	171.5	0	24.16716	96.33333	85	2	2	4	IOL	IOL	n	n	POAG	POAG	0	-0.07918	21	21	4	4	-10.7	-5.02
1314	82	M	222.5	0	24.52435	107.6667	82	2	0	2	lens	lens	n	n	other	other	0	-0.07918	25	26	2	2		
1316	73	F	385	0	27.84879	113.3333	86	3	2	5	IOL	IOL	n	n	other	other	-0.07918	-0.07918	23	23	3	3	-2.25	-3.56
1325	61	M	304	0	22.85714	115	73	3	2	5	lens	lens	n	n	other	other	-0.07918	0.221849	16	22	1	3	-1.02	-24.11
1327	61	F	429	0	23.12467	102.3333	79	3	2	5	lens	lens	n	n	POAG	POAG	-0.07918	0.522879	17	17	2	2	-2.26	-15.97
1328	85	M	260	0	22.58271	92.66667	62	3	2	5	lens	IOL	n	n	POAG	POAG	0.221849	0.221849	23	23	3	4		
1329	72	M	280	0	22.40818	99.66667	98	2	2	4	IOL	IOL	n	n	POAG	POAG	0	0	31	23	4	4	-8.1	-3.27
1338	69	F	330	0	20	97	78	3	2	5	IOL	IOL	y	y	EXG	EXG	-0.07918	0.09691	20	17	3	3	-12.92	-12.22
1339	69	M	373	0	25.18079	107	80	3	2	5	lens	IOL	n	n	other	other	0	-0.07918	13	14	3	3	-5.02	-24.64
1347	89	M	288.5	0	19.53125	108.3333	72	0	0	0	lens	IOL	n	n	other	other	2.886057	0.30103	52	33	0	3		-29.08
1348	72	F	510.5	0	19.17458	94.33333	72	3	2	5	lens	lens	y	n	EXG	POAG	0.09691	0.09691	27	21	3	2	-11.53	-2.16
1349	54	F	467	0	21.35931	92.33333	71	3	2	5	lens	lens	n	y	POAG	EXG	-0.07918	0.30103	25	50	5	5	-5.85	-22
1362	54	M	274.5	0	27.46481	128.3333	85	3	2	5	lens	lens	n	n	POAG	POAG	-0.07918	-0.07918	21	19	0	3	-4.33	-8.23
1364	66	F	389.5	0	20.02884	112.3333	76	3	2	5	lens	lens	n	n	POAG	POAG	-0.07918	0	19	19	3	3	-11.8	-28.36
1366	69	M	313.5	0	21.98989	105.6667	71	3	2	5	lens	IOL	y	n	EXG	other	0.154902	-0.07918	17	17	4	0	-11.01	0
1377	75	M	290.5	0	23.50781	87	71	3	2	5	lens	lens	n	n	other	other	0	0	32	25	2	2	-0.51	1.68
1378	82	M	136.5	0	17.52782	0		0	0	0	lens	IOL	n	y	other	other	2.886057	1.154902		12	2	2		-23.51
1379	46	M	253.5	1	28.07504	84	67	3	2	5	lens	IOL	n	n	other	other	-0.07918	-0.07918	19	48	1	5		
1380	75	F	304	0	28.53224	123.3333	58	2	2	4	lens	lens	n	n	other	other	0.09691	0	21	22	4	4	-7.3	-12.18
1410	85	F	580	0	25.96953	115.6667	66	2	2	4	IOL	IOL	n	n	POAG	POAG	-0.07918	0.045757	12	12	3	1	-6.53	-16.74
1411	83	M	111	0	15.6157	86	81	2	2	4	IOL	IOL	n	n	POAG	POAG	0.154902	0.154902	15	15	1	1	-27.97	-26.51
1412	55	M	267.5	0	24.34381	117	83	3	2	5	lens	lens	n	n	POAG	POAG	-0.07918	-0.07918	49	37	6	6	-3.48	-2
1420	62	M	169	0	21.19274	94.66667	74	3	2	5	lens	lens	n	n	POAG	POAG	0.69897	0.30103	52	42	0	0	-12.78	-6.06
1422	62	F	165	0	24.83576	97	84	2	2	4	lens	lens	n	n	POAG	POAG	1.154902	0.39794	24	20	1	1		
